# Selenium Nanoparticles Functionalized with Tumor-Targeting Peptides for Lung Cancer Therapy: A Comprehensive Review

**DOI:** 10.3390/ph19101601

**Published:** 2026-10-09

**Authors:** Areena Turk, Mohit Mishra, Vatsana Kasana, Tejveer Singh, Damandeep Kaur, Hardeep Singh Tuli, Tarique Sarwar, Arshad Husain Rahmani

**Affiliations:** 1 Translational Oncology Laboratory, Department of Zoology, Hansraj College, University of Delhi, Delhi 110007, India; 2 Department of Molecular Biology, Central University of Andhra Pradesh, Anantapur 515701, Andhra Pradesh, India; 3 Department of Homeopathy, Govt. Homeopathy Medical College & Hospital, Bharatpur 321001, Rajasthan, India; 4 University Center for Research & Development (UCRD), Chandigarh University, Gharuan, Mohali 140413, Punjab, India; 5 Centre of Excellence in Computational Research and Drug Discovery, Department of Bio-Sciences and Technology, Maharishi Markandeshwar Engineering College, Maharishi Markandeshwar (Deemed to Be University), Mullana, Ambala 133207, Haryana, India; hardeep.biotech@gmail.com; 6 Department of Medical Laboratories, College of Applied Medical Sciences, Qassim University, Buraydah 51452, Saudi Arabia; t.sarwar@qu.edu.sa

**Keywords:** non-small-cell lung cancer (NSCLC), selenium nanoparticles (SeNPs), green synthesis, phytochemicals, peptide-functionalized nanoparticles, targeted drug delivery, anti-cancer mechanisms, tumor targeting

## Abstract

Lung cancer is among the most fatal malignancies worldwide, with approximately 85% of diagnosed cases being non-small-cell lung cancer (NSCLC). Despite the considerable progress in surgical, chemotherapeutic, radiotherapeutic, and immunological treatments, the prognosis of NSCLC remains poor due to late-stage diagnosis, molecular heterogeneity, treatment resistance, recurrence, and systemic toxicity. Therefore, there is an urgent need to develop more effective and less toxic therapies, and cancer nanotechnology has emerged as a promising approach. Selenium nanoparticles (SeNPs) are a viable option for lung cancer treatment because of their unique physicochemical and biochemical properties. They can be used for targeted drug delivery into cancer cells, as selenium possesses inherent anti-cancer properties, such as the induction of reactive oxygen species (ROS), mitochondrial disruption, DNA damage, cell-cycle arrest, and modulation of several signaling pathways, including PI3K/Akt, MAPK, NF-κβ, STAT3, and p53, leading to cancer cell death and repression of tumor progression. Green synthesis of SeNPs using plant-derived phytochemicals is a sustainable strategy as it allows for simultaneous reduction and stabilization of nanoparticles, with an additional therapeutic phytochemical effect against cancer. Various phytochemicals, such as flavonoids, polyphenols, terpenoids, and alkaloids, have the ability to chelate metal ions and act as reducing agents and stabilizers in the synthesis of SeNPs. The antioxidant, anti-inflammatory, anti-proliferative, and anti-metastatic activities of phytochemicals, along with their ability to inhibit the overexpressed enzymes, e.g., matrix metalloproteinases, in cancer cells, could provide an additional therapeutic benefit. However, the application of SeNPs in practice is limited by their low targeting ability, which results in poor bioavailability due to fast clearance from the bloodstream, reduced tumor accumulation, and poor cellular uptake. Conjugation of SeNPs with tumor-targeting peptides could enhance their delivery into the cancer cells. There is a variety of cell surface receptors that are overexpressed in lung cancer cells and can be used for targeted drug delivery. The most common receptors are EGFR, integrins, gastrin-releasing peptide receptor, neuropilin-1, and transferrin receptor. The ligands for these receptors, including the GE11 peptide for EGFR (epidermal growth factor receptor), RGD for integrins, bombesin for GRPR, and iRGD for neuropilin-1, could be used for targeting, as well as cell-penetrating peptides, such as TAT. This review discusses the current knowledge on the application of functionalized SeNPs for lung cancer treatment, specifically synthetic methods, physicochemical properties, mechanisms of action, and targeting strategies. Particular attention is paid to the combination of the anti-cancer effects of selenium and phytochemicals and their synergistic impact on tumor cell death. In addition, the challenges of the application of SeNPs for lung cancer treatment and possible future developments for functionalized SeNPs as novel therapeutic nanocarriers are discussed.

## 1. Introduction

Lung cancer (LC) is still the foremost cause of death among all forms of cancer, accounting for a major global health problem. According to the latest cancer statistics (GLOBOCAN 2024), LC is one of the most common types of cancers that accounts for the highest number of cancer-related deaths despite the significant advances made in early detection and treatment [1]. NSCLC accounts for about 85% of all LCs and involves the dysregulated signaling pathways of EGFR, KRAS, PI3K/Akt, MAPK, and ALK. Although various therapies like surgery, chemotherapy, radiotherapy, targeted therapies, and immune checkpoint inhibitors have brought a great improvement in outcomes, their efficiency is often limited by late-stage diagnosis, heterogeneity of the tumors, multidrug resistance, off-target toxicity and recurrence [2]. Therefore, there is an immediate need for innovative treatment methods which could provide a more specific targeting of tumors without inducing any systemic toxicity. This makes nanomedicines a potential option [3].

SeNPs, from amongst the various nanomaterials that have been studied for potential applications in the treatment of cancers, appear to be especially promising due to their unique physical and chemical characteristics, high biocompatibility, and intrinsically possessed anti-cancer potential [4]. Selenium is an important trace element, which is a component of various seleno-proteins such as glutathione peroxidase and thioredoxin reductase proteins and which regulate cellular redox balance, antioxidant defenses and immunity [5,6]. In contrast to inorganic/organic selenium compounds, the advantages offered by SeNPs include higher bioavailability, better control over selenium release, increased biological stability, and significantly lower systemic toxicity [7]. Significantly, increasing scientific evidence suggests that SeNPs selectively produce cytotoxicity in cancerous cells but not in normal cells. The anti-cancer effect of SeNPs can be attributed to various mechanisms, which include ROS overproduction, mitochondrial depolarization, DNA damage, cell-cycle arrest, induction of apoptosis through the intrinsic pathway, inhibition of angiogenesis, as well as decreased tumor invasion and metastasis [7,8]. In addition, SeNPs affect the signaling pathways that play an important role in the development of lung cancer, such as the PI3K/Akt, MAPK, NF-κβ, STAT3, and p53 pathways [8,9,10,11]. Overall, these varied biological functions make SeNPs promising therapeutic candidates and drug carriers for the treatment of lung cancer.

While chemically synthesized SeNPs have great pharmacological potential, traditional strategies usually employ toxic reducing and stabilizing agents that may be detrimental to biocompatibility and limit their clinical application [12,13]. For this reason, green chemistry using natural phytochemicals extracted from plants is considered to be a promising and more biological approach [14]. There are various types of phytochemicals, such as flavonoids, phenolic acids, alkaloids, terpenoids, tannins, saponins, and anthocyanins, that can act as reducing, capping, and stabilizing agents for nanoparticle synthesis without toxic materials [15,16,17]. In addition to being used for nanoparticle synthesis, these natural compounds can provide additional beneficial effects by promoting colloid stability, preventing aggregation, facilitating cellular uptake, extending circulation in blood, and improving biocompatibility. Most importantly, phytochemical-functionalized SeNPs exhibit synergy in the anti-cancer properties due to the interaction of selenium and plant molecules, resulting in better pro-oxidative cytotoxicity and anti-proliferative, anti-inflammatory, and anti-metastatic activities than unperturbed SeNPs [17,18,19].

**Figure 1:**
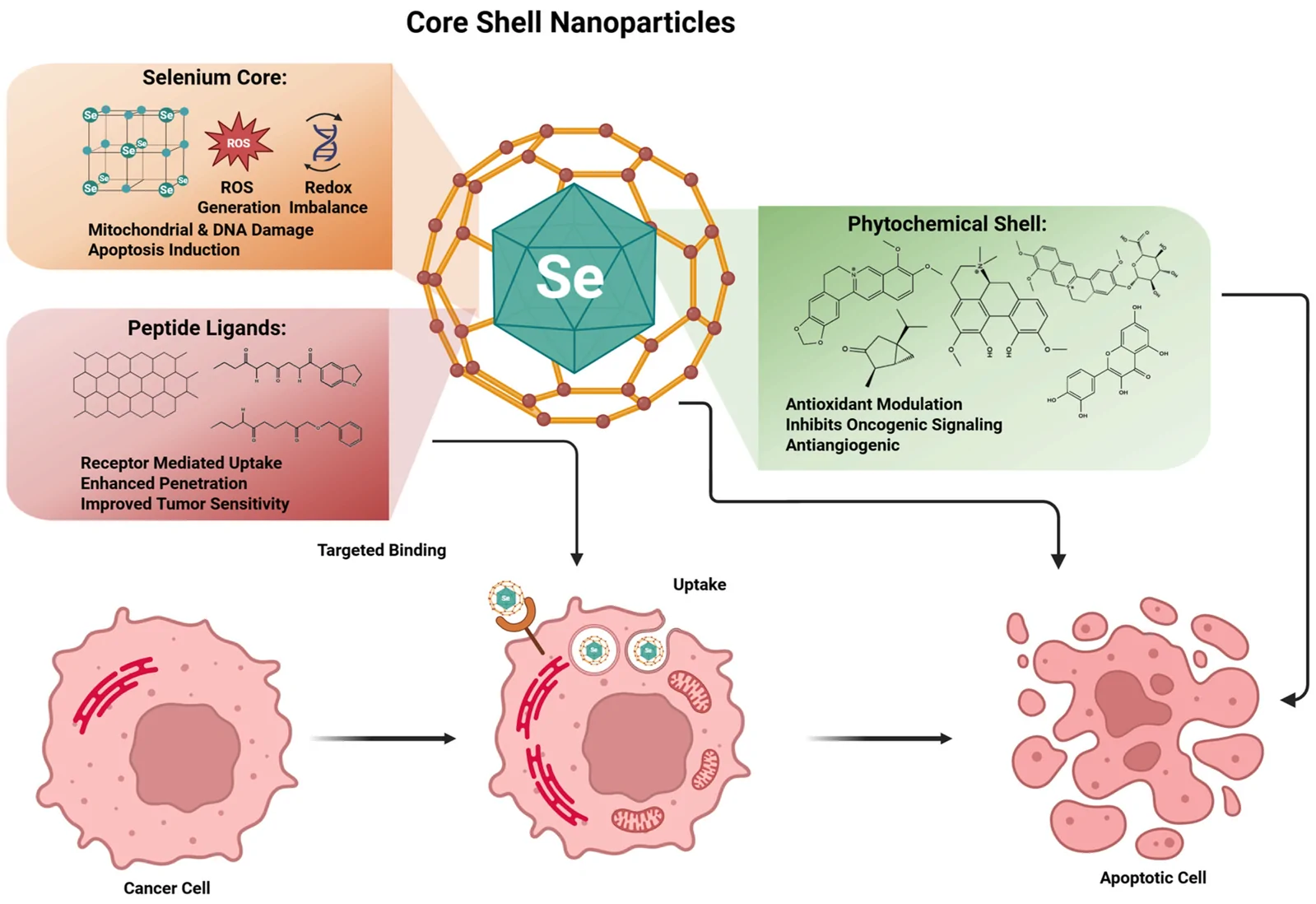
**Synergistic anti-cancer mechanism of peptide-directed phytochemical-coated SeNPs.** The selenium core, phytochemical shell and surface-functionalized peptide ligands—the conjugation of these three can facilitate receptor-mediated tumor targeting, improve cellular uptake, enhance tissue penetration, and increase therapeutic selectivity. The combined actions of the selenium core, phytochemical coating, and peptide ligands synergistically inhibit tumor growth and promote apoptotic cancer cell death while minimizing off-target toxicity. Created in BioRender. RAHMANI, A. H. (2026). https://biorender.com/n7lfm28, accessed on 30 September 2026.

In spite of significant achievements in nanotechnology, issues such as nonspecific biodistribution, rapid uptake by mononuclear phagocytes, poor tumor penetration, and insufficient cell uptake still limit the efficacy of nanotherapeutics [20]. Peptide functionalization has proven to be one of the most efficient approaches to overcome the mentioned limitations by facilitating the receptor-mediated and target-specific delivery of drugs (Figure 1) [21,22]. The use of peptides has several advantages over conventional targeting ligands; they include a low molecular weight, high receptor specificity, deep tissue penetration, low immunogenicity, easy chemical synthesis, and simple conjugation to the nanoparticle surface [23]. A number of different targeting peptides, such as RGD, GE11, TAT, bombesin, iRGD, as well as various receptor-specific peptides, show significant promise in the specific targeting of overexpressed receptors in lung cancer cells, including the epidermal growth factor receptor (EGFR), integrins, gastrin-releasing peptide receptor (GRPR), neuropilin-1, and transferrin receptor. Conjugation of peptides significantly increases the efficiency of receptor-mediated uptake, accumulation in tumors, drug delivery to the cells, and drug efficiency while decreasing off-target toxicity [24]. Combining peptide targeting with phytochemical-coated SeNPs results in the development of a multipurpose nanostructure with selenium-mediated inherent anti-cancer effects [23], phytochemical-mediated therapeutically enhanced effects, and high tumor-targeting efficiency, providing a perspective approach for overcoming multidrug resistance in lung cancer [25].

## 2. Tumor Microenvironment (TME)

Lung cancer TME includes tumor cells, cancer-associated fibroblasts (CAFs), endothelial cells, immune cells, stromal fibroblasts, extracellular matrix (ECM) components, inflammatory mediators, and signaling molecules, which coordinate tumor initiation, development, metastasis, and treatment efficacy [26]. There is mounting evidence supporting the idea that mutual interactions between cancer cells and the environment around them play an equally significant role in disease progression as do internal genetic mutations. In the TME, these interactions cause tumor immuno-evasion, angiogenesis, metastasis, and resistance to chemotherapy, radiation therapy, and targeted therapy [27,28,29]. CAFs contribute to ECM remodeling due to increased collagen deposition and the activity of matrix-remodeling enzymes, leading to increased tissue rigidity, tumor fibrosis, and chemoresistance [30]. Moreover, ECM itself forms a large physical barrier to nanoparticle penetration into the tumor tissues [31]. The involvement of tumor-associated macrophages (TAMs), predominantly the M2 subtype, in immune suppression, angiogenesis, and cancer progression is facilitated by the secretion of pro-tumorigenic cytokines and growth factors [32]. Additionally, tumor-derived exosomes act as important mediators of intercellular communication, delivering proteins, nucleic acids, and lipids from tumor cells to stromal cells and thus facilitating pre-metastatic niche formation, immunomodulation, and treatment resistance [26]. Overall, these stromal and immune components form a complex microenvironment that, on the one hand, contributes to disease development but, on the other hand, impedes nanoparticle penetration into tumors and affects therapeutic efficacy. Therefore, peptide-functionalized selenium nanoparticles designed for selective interaction with TME-related receptors present a promising tool for improving nanoparticle infiltration into tumors [26,31].

Hypoxia is typical for fast-growing lung tumors caused by insufficient vascularization and oxygenation of growing tumors. Reduced oxygen concentration leads to HIF-1α stabilization that transcriptionally regulates genes associated with angiogenesis, glycolytic metabolism, invasion, metastasis, and survival, such as VEGF [33]. Hypoxia also helps in maintaining cancer stem cells and causes resistance to chemotherapy, radiation therapy, and some targeted therapies, and thus negatively affects clinical outcome [34,35]. However, it should be noted that the proposed targeting of hypoxic tumor regions by peptide-functionalized SeNPs is still only a prospective approach, and the success of such a treatment concept should be validated by identification of hypoxia-associated receptors and proof of concept in NSCLC models [36]. It is also assumed that such targeting may be achieved due to the enhanced affinity of peptides to hypoxic tumor regions; however, it should be validated experimentally [37].

Lung carcinogenesis and tumorigenesis are greatly influenced by oxidative stress [38]. Chronic exposure to cigarette smoke, environmental toxins, mitochondrial defects, and oncoprotein signaling increases the production of ROS inside the cell [39]. The physiological level of ROS is involved in cell signaling; however, increased levels cause damage to DNA, instability of the genome, aberrant growth, and tumor development [40]. On the other hand, the excessive amount of ROS surpasses the ability of antioxidant defenses and causes mitochondrial malfunction, apoptosis, and cell death. Therefore, lung cancer cells maintain redox homeostasis with the help of an enhanced antioxidant system to ensure their survival [41]. Selenium nanoparticles (SeNPs) have special features associated with redox modulation that make it possible to target redox homeostasis in tumor cells and induce apoptosis by generating higher amounts of ROS than the cell can tolerate, whereas the side effects on healthy cells are lower [42]. A dysregulated redox state in the tumor microenvironment becomes an excellent target for SeNP treatment [43]. The peptide-based delivery of SeNPs makes it possible to generate ROS specifically in tumors and to trigger apoptosis of cancer cells [44].

Angiogenesis is involved in the tumor growth and metastasis process by providing the tumor tissues with oxygen and nutrient supplies [45]. In non-small-cell lung cancer, the VEGF/VEGFR signaling pathway is one of the most important controllers of tumor angiogenesis and therefore an attractive target for therapy [46]. Nevertheless, the vasculature in tumor tissues is structurally abnormal, permeable, and disorganized, causing heterogeneous distribution of perfusion and low efficacy of drug delivery [47]. Thus, anti-angiogenic agents that target VEGF/VEGFR signaling have been incorporated into the treatment protocols of NSCLC [48]. Selenium nanoparticles delivered to the tumor tissue via peptide ligands that target VEGFR or angiogenic endothelial cells might dramatically increase the delivery of selenium nanoparticles in highly angiogenic tumors [48]. Such targeted nanostructures could be used not only for anti-angiogenic but also drug delivery purposes [49].

Immune suppression inside the tumor microenvironment is an important contributor to the development of lung cancer and drug resistance [50]. Tumor cells escape immune system attack via various pathways, such as PD-L1 expression, induction of regulatory T cells (Tregs), generation of myeloid-derived suppressor cells (MDSCs), and activation of tumor-associated macrophages into immunosuppressive M2 macrophages [51]. Cytokines produced by these cells include IL-6, TGF-β, and IL-10. They inhibit the cytotoxic function of T cells, induce chronic inflammation, support tumor growth, and increase their metastatic capacity [52]. The effectiveness of drugs blocking the PD-1/PD-L1 pathway is evidence for the importance of overcoming immune suppression in NSCLC [53]. Peptide-conjugated selenium nanoparticles can offer possibilities for overcoming immune suppression inside the tumor microenvironment and targeted therapy against immune checkpoints and tumor-associated immune cells [38]. In this way, it can be assumed that peptide-conjugated selenium nanoparticles might enhance the immune response to cancer and simultaneously increase the efficiency of immunotherapy of NSCLC [54].

Apart from hypoxia and immune suppression, several other stromal elements such as CAFs, the ECM, tumor-associated macrophages (TAMs), and tumor-derived exosomes have been identified as crucial determinants of lung cancer progression [55]. CAFs are responsible for ECM remodeling and fibrosis, whereas the dense ECM network is characterized by the inability of nanoparticles to enter tumor tissues [55]. The activation of M2-polarized TAMs results in angiogenesis, immune suppression, and metastatic progression due to their ability to secrete cytokines [56]. In addition, tumor-derived exosomes have been shown to provide intercellular communication and pre-metastatic niche formation as well as to cause drug resistance [57]. Thus, all these stromal elements should be targeted using peptide-functionalized selenium nanoparticles [4].

Thus, the TME serves not only as a significant biological barrier but also as a promising therapeutic target for non-small-cell lung cancer [58]. Hypoxia, oxidative stress, abnormal angiogenesis, immune suppression, stromal remodeling, and ECM deposition contribute to the development of tumors and drug resistance and, in turn, limit the effectiveness of anti-cancer therapy [59,60]. The combination of peptides and selenium nanoparticles could serve as a multi-targeting tool since peptide-mediated targeting allows for the selective targeting of tumor-associated receptors, while selenium nanoparticles can increase tumor penetration, affect oxidative stress, inhibit angiogenesis, reprogram immunosuppressive tumor microenvironments, and provide more efficient intracellular drug delivery [35,61].

## 3. Targets Relevant to Peptide-Mediated Therapy

Due to its overexpression and presence of mutations in non-small-cell lung cancer (NSCLC), EGFR has been extensively characterized and explored as a molecular target for the development of treatments for lung cancer [62,63]. EGFR-targeted peptides such as GE11 can be used to functionalize therapeutic nanoparticles, enabling highly specific and targeted delivery of the therapeutic nanoparticle directly into the EGFR-overexpressing tumor cells [64,65].

Although EGFR-targeted TKIs have achieved success in clinical settings, acquired resistance is frequently encountered and is caused by secondary mutations (e.g., T790M and C797S) or bypass signaling mechanisms, such as MET amplification and the activation of alternate receptor signaling [66,67]. EGFR-targeted peptide-conjugated SeNPs could be applied as a versatile delivery vehicle for targeted therapy in EGFR-mutant NSCLC [68,69]. EGFR-targeting peptide–SeNP conjugates are able to deliver TKIs, siRNAs, CRISPR/Cas gene-editing systems, and combination treatments effectively into the tumor cells [69]. Targeted delivery enhances the intracellular concentration of drugs, minimizes toxicity of systemic administrations, and shows great potential in addressing drug resistance in EGFR-mutant NSCLC [70].

Another ErbB receptor family member, called human epidermal growth factor 2 (HER2), is also important in the promotion of lung cancer via activation of PI3K/Akt and MAPK pathways [71]. HER2-targeted peptide ligands have also proven effective at increasing the accumulation of theranostic nanoparticles in HER2-positive tumors and the selective delivery to these tumors [72]. HER2 mutations are present in 2–4% of NSCLC, and these are commonly associated with aggressiveness, poorer prognosis, and resistance to current therapies [73,74]. Peptides directed to HER2 promote a significant increase in the accumulation of SeNPs in HER2-positive tumors, allowing improved intracellular drug delivery and increased efficiency of the targeted therapy [75]. Hence, SeNPs loaded with peptides targeted to HER2 have demonstrated potential as precision medicine targeting lung cancers with HER2 alterations [76].

Integrin receptors that are highly expressed on both tumor and angiogenic endothelial cells govern adhesion, migration and invasion of tumor cells, and also control the process of angiogenesis [77]. RGD-containing peptides have been found to target integrin receptors, resulting in tumor-targeted delivery of nanoparticles in lung cancer treatment [78]. In lung cancer treatment, targeting of the integrin receptors increases targeted specificity and disrupts both angiogenesis and the spread of tumor metastases [79]. Integrin V3 and V5 receptors are particularly abundant on both tumor and angiogenic endothelial cells, making them ideal targets for peptide-based nanomedicine [80]. Functionalization of SeNPs with the RGD or iRGD peptides significantly improves tumor penetration, enhances receptor-mediated endocytosis, inhibits tumor angiogenesis and permits highly efficient intracellular drug delivery. Furthermore, integrin-targeting SeNPs reduce tumor invasion and metastatic spreading, improving the targeting specificity and minimizing systemic toxicity [24,81].

The vascular endothelial growth factor receptor (VEGFR) a target that has been widely pursued to develop innovative cancer therapy, since this receptor plays a vital role in the regulation of tumor angiogenesis has also emerged as an important therapeutic target [35,56]. The peptide-conjugated nanoparticles targeted to VEGFR were able to localize in the tumor vascular bed and deprive the TME of nutrients by inhibiting blood vessel development (anti-angiogenesis) [82]. Adaptor proteins that link different receptor tyrosine kinases to the Ras/MAPK signaling cascades, namely GRB2 and SHC, have also been explored as targets for peptide-directed nano-therapies. GRB2 facilitates coupling between the receptor and Ras/MAPK cascades, and SHC is the primary link responsible for the signaling pathways regulating cellular survival and proliferation; thus the development of novel peptide-based nano-therapies that inhibit the intracellular signaling adaptor proteins may lead to successful treatment of receptor-targeting therapy-resistant lung cancer [83,84]. In addition to the inhibition of tumor angiogenesis, the VEGFR-targeting peptide-functionalized SeNPs provide a convenient platform for highly specific delivery of anti-angiogenic agents, siRNA, gene-editing molecules, or a combination of drugs directly to tumor vessels, which will achieve the overall therapeutic effect by interrupting neovascularization and reducing blood supply [84,85].

Adaptor protein targets in EGFR-mediated signaling pathways (GRB2 and SHC) have been applied to develop peptide-directed nano-therapies. GRB2 mainly connects active receptor tyrosine kinases to the Ras/MAPK signaling pathway, and SHC mainly conveys downstream signals related to cell proliferation and survival [86,87]. Targeting intracellular signal adaptor proteins with peptide-based nanotherapeutics may provide a new pathway for overcoming resistance in receptor-targeted therapy [88]. Peptide-functionalized SeNPs can effectively serve as platforms for delivery of siRNA, antisense oligonucleotides, and small molecule inhibitors to intracellular adaptors such as GRB2 and SHC [89].

Simultaneous inhibition of those signaling mediators, thus, could suppress multiple oncogenic pathways downstream of RTKs (such as Ras/MAPK and PI3K/Akt pathways), thus repressing tumor growth, preventing the occurrence of acquired drug resistance, and promoting therapeutic efficacy [90,91]. Another critical targeting molecule regarding the progression of lung cancer is the signal transducer and activator of transcription 3 (STAT3) [92]. Persistent activated STAT3 has been found in all types of non-small-cell lung cancer (NSCLC), which provides a mechanism for tumor aggressiveness and therapy resistance by promoting angiogenesis, suppressing immune responses and resisting apoptosis [61]. Targeting STAT3 with peptides has been exploited as a promising therapeutic approach to inhibit tumor development and enhance the efficiency of conventional chemotherapy and other anti-cancer agents. By targeting STAT3, peptide-functionalized SeNPs can suppress immune evasion, reduce inflammation, overcome chemoresistance and restore apoptotic signaling pathways [35,93]. The major molecular targets implicated in NSCLC progression and their potential relevance to peptide-directed SeNP-based therapeutic strategies are summarized in Table 1.

## 4. Selenium Nanoparticles: Synthesis and Properties

Synthesis methods for selenium nanoparticles (SeNPs) have become increasingly popular in the nanotechnology arena and are considered the best option in terms of reduced toxicity, biological properties, and treatment potential in several areas: cancer, antimicrobial, antioxidant, and drug-delivery applications [36,61]. SeNPs can be produced by several different means that provide control over physicochemical characteristics and biological functions of the manufactured particles [61]. They are generally produced via three broad categories.

### 4.1. Chemical Synthesis

Chemical synthesis is generally regarded as the most preferred approach for fabricating SeNPs since it provides easy synthesis, replicability, and produces perfectly sized or shaped particles [36]. Another common method of SeNPs synthesis involves the chemical reduction of selenium salts (such as sodium selenite (Na_2_SeO_3_) or selenium dioxide) using various reducing agents (ascorbic acid, hydrazine hydrate, sodium borohydride (NaBH_4_), glucose, ethylene glycol) to produce the reduced form of selenium (Se^0^) [101,102]. Furthermore, to prevent these newly created SeNPs from agglomerating or to stabilize the created SeNPs, it is common practice to introduce stabilizers/capping agents such as polyvinyl alcohol (PVA), chitosan, polyethylene glycol (PEG) or bovine serum albumin (BSA) [101].

Typically, the selenium nanoparticle synthesis involves the reduction of Se ions in an aqueous medium (water) with control of the following factors: temperature, pH value, stirring, etc.

Formation of the SeNPs often appears with an observable color change from colorless to orange-red, which can be attributed to the presence of surface plasmon resonance [103]. Listed below are the advantages of using chemical synthesis for synthesizing SeNPs: short synthesis time, good yields, controlled size and shape, and easy scale-up. On the other hand, some disadvantages are that the toxic chemicals employed and hazardous waste create potential contaminants that can alter surfaces, which can cause restrictions in biomedical applications [104]. However, various research papers in recent years have focused on the optimization of conditions for synthesizing more uniform particle-size distributions, increasing drug-loading efficiency, and minimizing the level of toxicity for SeNPs in terms of reducing the amount of reactant (e.g., reducing agents), the amount of solvent, temperature, and reaction time [105]. The optimization of SeNP synthesis processes involves the control of precursor concentration, reducing-agent-to-selenium molar ratio, stabilizer or capping agent concentration, pH, temperature, stirring conditions, reagent addition order, and reaction duration [36]. These parameters directly impact the mechanisms of nucleation and growth and, therefore, the resulting NP size, size distribution, morphology, surface chemistry, colloidal stability, and yield. In the case of peptide functionalization, the selection of the appropriate conjugation or crosslinking chemistry is equally important. EDC/NHS chemistry is optimal for carboxyl–amine coupling, while maleimide-thiol chemistry can be utilized for peptides containing reactive thiol groups; alternatively, click-chemistry strategies can be applied in the case of bio-orthogonal conjugation [35]. However, it should be noted that the molar ratios of the reagents and the reaction duration should be optimized for each formulation, as changes in these conditions can affect NP growth and the efficiency of peptide coupling [19]. To enable the replication of the results, future studies should report the concentrations of the used precursors and reagents, molar ratios, pH, temperature, reaction time, stirring conditions, isolation/purification procedure, and storage environment in detail. Furthermore, newer synthesis approaches, including microwave-assisted synthesis and hydrothermal and solvothermal methods, are actively explored to generate nanoparticles with precisely designed characteristics [106].

### 4.2. Green Synthesis

Ever since the discovery of the concept of ‘green’ technology, green synthesis has emerged as an environmentally friendly and sustainable process that can be an alternative to the chemical synthesis of materials by utilizing materials found in nature, such as polysaccharides, flavonoids, phenolics, proteins, or plant extracts, as reducing and stabilizing agents [107,108]. Plant extracts utilized in the SeNPs synthesis are: *Allium sativum* (Garlic), Aloe Vera, *Camellia sinensis* (Green Tea), *Azadirachta indica* (Neem), and *Berberis aquifolium* [109,110,111,112]. These extracts contain phytochemicals that play a dual role; they are involved in the reduction of Se ions to Se and are involved in encapsulating the surfaces of SeNPs. This additional shell makes SeNPs much more stable and biocompatible [113]. There are several benefits offered by this method: it is an eco-friendly process, utilizes fewer hazardous elements, and has relatively low-cost high biocompatibility [114]. The inclusion of bioactive substances along with SeNPs can lead to synergistic antioxidant, anti-cancer, and antimicrobial activity [115]. On the other hand, green synthesis has its limitations in high batch-to-batch variability [116]. The products are not highly reproducible, size and shape are not so controllable, and phytochemical components may vary; nonetheless, the advantages of green synthesis are being increasingly valued for producing SeNPs for biomedical applications due to their low toxicity and their effectiveness [117].

### 4.3. Physicochemical Properties and Biological Potential of Phytochemical-Coated Selenium Nanoparticles

The therapeutic potential of SeNPs is largely dependent on their physicochemical characteristics such as size, shape, surface charge, colloidal stability, and surface chemistry, which greatly affect cell uptake, biodistribution, drug-loading efficiency and biological action [118,119]. Nanoparticles less than 100 nm may possess the tendency to accumulate in tumors due to enhanced permeability and retention (EPR) effects, whilst nanoparticles that are more spherical are typically more stable colloids and also more equally taken up by cells [120]. Zeta potential influences a nanoparticle’s stability, ability to interact with cell membranes, circulation half-life, and cellular uptake, whilst surface chemistry affects stability and aggregation [121].

Due to the large surface-to-volume ratio and tunable chemistry, SeNPs are highly effective for loading or conjugating a variety of therapeutic agents such as phytochemicals, peptides, proteins, and nucleic acids in an attempt to provide controlled release and improve bioavailability [122,123].

A rational nanoscale engineering strategy should focus on combining the synthesis route with subsequent surface functionality rather than treating these as discrete processes [36]. While chemical synthesis provides relatively higher control over the reaction conditions and physicochemical attributes, green synthesis routes can provide biofunctional reducing and capping components that require tighter standardization of the extract composition since phytochemical diversity can significantly impact the yield of the desired nanomaterial in terms of size, shape, and reproducibility [124]. Phytochemical-based surface coating should offer enhanced stabilization while leaving the necessary functional groups exposed for subsequent peptide conjugation and receptor recognition The reported data on the phytochemical functionalized SeNP systems, the cancer cell lines tested, their molecular/cellular mechanisms, and therapeutic outcomes are summarized in Table 2, highlighting their potential in NSCLC therapy [19]. During the process of peptide conjugation, the ligand density, orientation, conjugation chemistry, and linker attributes should be optimized to avoid steric hindrance and ensure that targeting properties are not compromised [61]. Finally, achieving superior colloidal stability will necessitate a higher degree of control over the size and PDI, surface charge, capping agent coverage, and resistance to aggregation under physiological conditions. Thus, the colloidal stability should not only be assessed in water or aqueous media, but also in the presence of serum proteins, where the altered surface properties can have a significant impact on nanoparticle behavior.

Comprehensive characterization of the peptide-functionalized SeNPs should go beyond reporting the mean particle size. The polydispersity index (PDI) should also be reported to characterize the distribution of sizes, while information about the morphology, shape, and uniformity of the particles can be obtained by transmission or scanning electron microscopy [123]. Zeta potential and temporal dynamic light scattering measurements can elucidate the colloidal stability, while incubation in serum or plasma can challenge the particles and disclose possible protein corona formation and associated changes in particle size or morphology [123]. Similarly, chemical and surface stability should be evaluated by following the selenium content or oxidation state, the state of the surface coating, peptide loading and retention, and the uniformity of functionalization. For self-assembled systems, the kinetics of assembly/disassembly should be determined at physiologically relevant and tumor-mimicking conditions and in the presence of denaturing agents if relevant (e.g., changing pH, reducing agents, or specific enzymes). Finally, batch-to-batch consistency should be ascertained by analyzing a set of particles produced in different batches for predetermined quality attributes, such as size, PDI, morphology, zeta potential, selenium content, surface composition, and peptide loading. Overall, standardized evaluation and reporting of such characteristics will contribute to consistent results and facilitate comparisons between different formulations and studies.

Biologically synthesized SeNPs or phytochemical-coated SeNPs have enhanced compatibility, antioxidant activity and stability while reducing toxicity due to the capping action of biomolecules of plants [15]. SeNPs have also demonstrated pronounced activity against cancer via the inducement of reactive oxygen species (ROS)-mediated cell apoptosis, DNA damage, cell-cycle arrest, mitochondrial dysfunction, tumor cell invasiveness, and metastasis [4]. However, the currently available traditional and phytochemical-coated SeNPs mostly utilize passive tumor accumulation to enhance their presence in tumors, thus leading to low tumor specificity, heterogeneity within tumors, unwanted uptake by normal cells and less therapeutic effect [125]. Therefore, the peptide-targeted SeNPs were designed in which specific tumor homing peptides would be conjugated to the surface of SeNPs to actively target tumors by means of receptor-mediated endocytosis, leading to greater tumor localization and cellular uptake and higher precision therapy with reduced toxicity [49].

**Table 2:** Phytochemical-functionalized selenium nanoparticles investigated for anti-cancer activity and their relevance to lung cancer therapy: phytochemical-functionalized SeNP systems, the cancer models evaluated, their principal molecular and cellular mechanisms, and the reported therapeutic outcomes, highlighting their potential application in NSCLC treatment.

S. No.	Phytochemical/Biological Coating	SeNP Formulation	Cancer Model	Major Mechanism	Therapeutic Outcome	Relevance to the Present Review	Ref.
1.	Curcumin	Curcumin-functionalized SeNPs	Lung cancer/NSCLC models	ROS generation, mitochondrial dysfunction, modulation of NF-κβ and PI3K/AKT signaling	Enhanced apoptosis and suppression of proliferation	Directly relevant; provides rationale for combining phytochemical activity with peptide-mediated targeting	[126,127]
2.	Quercetin	Quercetin-functionalized SeNPs	Lung cancer	ROS generation, mitochondrial dysfunction, inhibition of migration and survival signaling	Increased cytotoxicity and reduced migration	Directly relevant to phytochemical-coated SeNP therapy	[128]
3.	Resveratrol	Resveratrol-functionalized SeNPs	Cancer models	PI3K/AKT and STAT3 modulation, mitochondrial apoptosis	Increased tumor-selective activity	Supports the multifunctional activity of phytochemical-coated SeNPs	[129]
4.	Plant-derived phytochemical extracts	Biosynthesized/plant-functionalized SeNPs	A549 and other NSCLC models	ROS-mediated apoptosis, caspase activation, cell-cycle modulation	Reduced cell viability and enhanced apoptosis	Demonstrates the broader potential of phytochemical-mediated SeNP functionalization	[128,130,131]

## 5. Peptide Functionalization of Selenium Nanoparticles (SeNPs)

The use of peptides to functionally modify the surfaces of selenium nanoparticles (SeNPs) has become an attractive means to enhance the specificity, cellular uptake and therapeutic effectiveness of nanomedicine [58]. SeNPs have anti-cancer, antioxidant and immunomodulatory properties in their own right, while peptide ligands provide active targeting abilities to cancer cells [80]. Functionally modifying SeNPs with peptides improves their stability in biological fluids, prolongs their circulation time, facilitates receptor-mediated internalization and decreases nonspecific toxicity to healthy tissue [132]. Due to these advantages, peptide-directed SeNPs are being studied more and more as targeted drug delivery systems for both cancer therapy and theranostic applications [133].

### Peptides as Targeting Ligands

**Figure 2:**
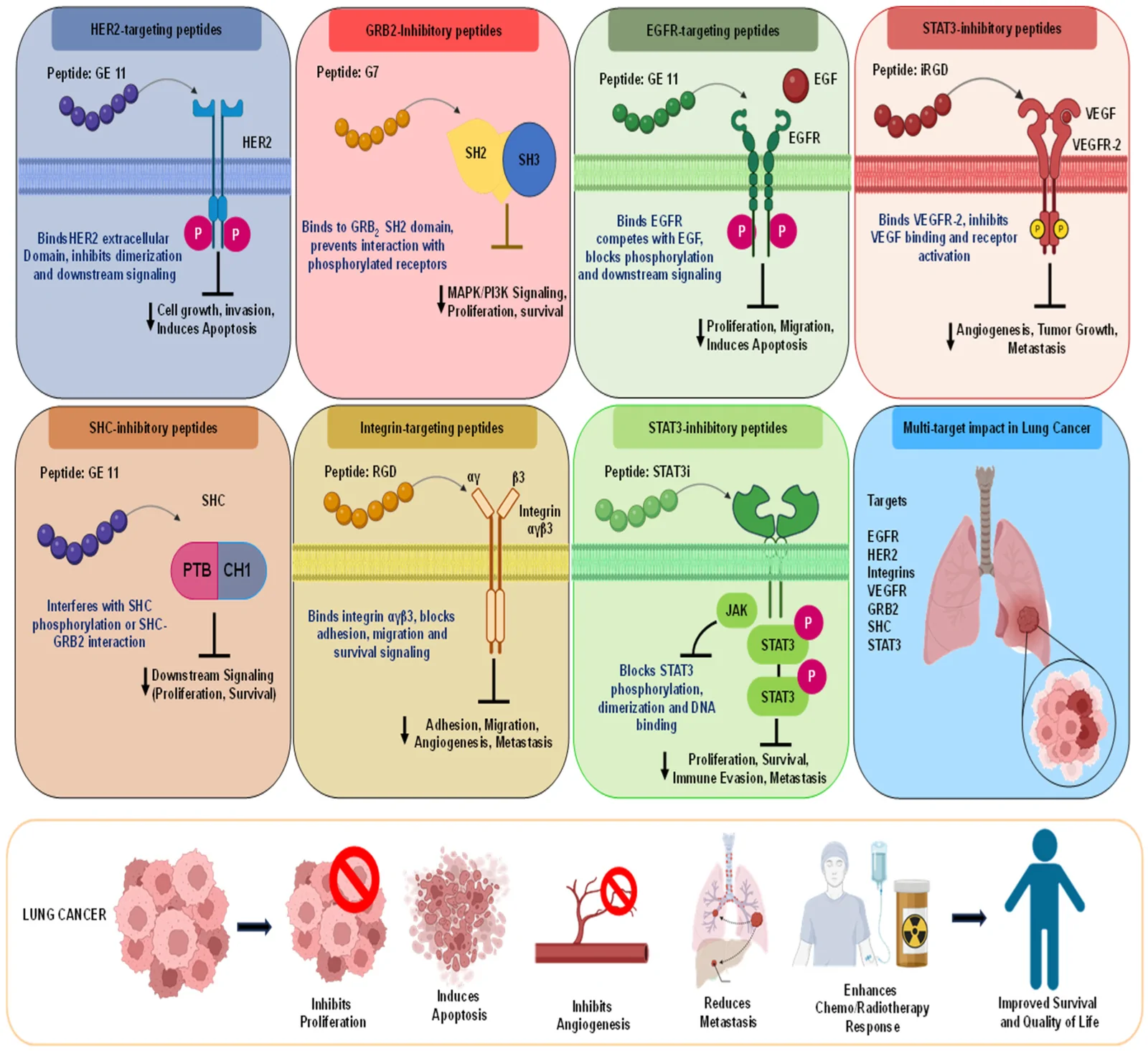
**Molecular targets and mechanisms of peptide-directed nanotherapeutics in lung cancer.** Peptide ligands selectively target key oncogenic receptors and signaling proteins, including EGFR, HER2, VEGFR-2, integrins (αvβ3/αvβ5), GRB2, SHC, and STAT3, to inhibit receptor activation and downstream pathways involved in tumor proliferation, survival, angiogenesis, migration, invasion, immune evasion, and metastasis. By enabling receptor-specific targeting and enhanced cellular uptake, peptide-functionalized nanocarriers improve therapeutic selectivity, overcome tumor heterogeneity and drug resistance, and enhance the overall efficacy of lung cancer treatment. Downward arrow (↓) denotes decreased/downregulation. Created in BioRender. RAHMANI, A. H. (2026) https://BioRender.com/spx5gaq, accessed on 28 September 2026.

Peptides are short chains of amino acids that can bind specifically to receptors, transporters, or other membrane components that are overexpressed on tumor cells [134]. They have several advantages over antibodies, including lower immunogenicity, simpler synthesis, better tissue penetration, and the ability to be easily chemically modified [135]. The small size of peptides enables them to be efficiently conjugated to the surface of nanoparticles without dramatically influencing the physicochemical properties of the nanoparticle itself [136]. Once conjugated, peptide ligands can direct SeNPs to their target (tumor) tissue via receptor-mediated endocytosis, thereby enhancing their intracellular accumulation and resulting therapeutic effect [137]. Some of the peptides used till now are discussed in this review, including the RGD peptide, GE11 peptide and Bombesin Peptide; the Arg-Gly-Asp (RGD) peptide represents one of the most frequently studied receptor-targeting ligands in cancer nanomedicine today [138]. RGD exhibits specificity towards integrin αvβ3 receptors, which regulate important processes involved in tumor angiogenesis, invasion, and metastasis [139]. RGD-modified nanoparticles show a greater attachment to the endothelium of the tumor and accumulate to a greater extent in the tumor tissue than non-modified nanoparticles [140]. In this regard, incorporation of RGD onto the surface of SeNPs may facilitate targeted delivery and have the potential to enhance the anti-angiogenic properties associated with anti-cancer drugs [141]. The targeting of therapeutics through the use of RGD is particularly applicable to breast cancer, glioblastoma, melanoma, and cervical cancer [139]. The GE11 (YHWYGYTPQNVI) peptide is a synthetic version of the epidermal growth factor (EGF), which has a high affinity for the epidermal growth factor receptor (EGFR), but does not have the same mitogenic activity as EGF [142]. In many cancers, including cervical, colorectal, breast, and non-small-cell lung cancers, there is an overproduction of EGFR [143]. The addition of the GE11 peptide to selenium nanoparticles (SeNPs) allows them to selectively bind to EGFR-positive cells and be internalized through receptor-mediated mechanisms [144]. These peptides with their mechanism are summarized in Table 3. This strategy improves the intracellular delivery of therapeutic cargo while not activating tumor growth pathways [19]. Bombesin is a peptide ligand that binds to gastrin-releasing peptide receptors (GRPRs), which are overexpressed in prostate, breast, pancreatic, and lung cancers [145]. Bombesin-conjugated nanoparticles have demonstrated efficient receptor-mediated targeting and enhanced tumor localization [146]. Functionalization of SeNPs with bombesin can improve uptake by GRPR-positive tumors and enable targeted drug delivery with decreased systemic toxicity [147]. The mechanism of action of different ligands and their inhibition via peptide are illustrated in Figure 2.

## 6. Peptide Conjugation Strategies

The most frequently used method for functionalizing nanoparticles with peptides is covalent conjugation, as it provides a strong and stable bond under physiological conditions [158]. Peptides that contain amino groups (-NH2), thiol groups (-SH), or carboxyl groups (-COOH) are usually connected to a nanoparticle surface using one of three methods: carbodiimide-mediated coupling (EDC/NHS chemistry), maleimide-thiol reactions, or click chemistry [159,160,161]. Covalent attachment offers the benefit of providing stability through longer circulation times within the body, preventing premature dissociation (from the nanoparticle) when the peptide is administered systemically [159]. Electrostatic adsorption relies on non-covalent attractive forces between opposite charges of both the peptide and the nanoparticle [162]. For example, a positively charged peptide will adsorb onto a negatively charged selenium nanoparticle (SeNP) surface via electrostatic attraction (non-covalent) [163]. This technique is relatively straightforward, inexpensive, and requires no complex chemical modifications [164]. Unfortunately, electrostatic attractions are usually weaker than covalent bonds; therefore, if the physiologic conditions surrounding the nanoparticle change, electrostatic attractions may no longer exist, and peptides may desorb from the nanoparticle surface [162,164]. Linker chemistry or linker molecules (e.g., polyethylene glycol [PEG], disulfide bonds, hydrazone linkers, and enzyme-cleavable spacers) are generally employed to provide improved orientation and functionality of the peptide attached to the nanoparticle [165]. For example, using PEG as the linker molecule reduces steric hindrance and improves the circulation time of the nanoparticle in the body, while stimuli-responsive linkers allow controlled release of the peptide from the nanoparticle when subjected to a specific condition (e.g., acidic pH, redox conditions, or enzymes produced by cancer cells) [166]. Therefore, linker chemistry plays a significant role in improving therapeutic performance and targeting efficiency [167].

## 7. Cellular Uptake and Internalization

### 7.1. Receptor-Mediated Endocytosis

The uptake of peptide-functionalized selenium nanoparticles (SeNPs) by cancer cells is primarily through endocytosis and is mediated by receptor binding [168]. Targeting peptides such as RGD, GE11, and Bombesin specifically target receptors overexpressed in tumor cells detailed as follows: RGD binds integrins (αvβ3), GE11 binds the epidermal growth factor receptor (EGFR), and Bombesin binds the gastrin-releasing peptide receptor (GRPR) [169]. Once bound, each of these ligands activates either clathrin-dependent or caveolae-dependent endocytosis, leading to efficient internalization of the SeNPs [170]. It has been shown that nanoparticles modified with RGD accumulate to a significantly greater extent in integrin-positive tumor cells compared to those that are not targeted [132]. Similarly, GE11-functionalized nanocarriers had greater uptake in EGFR-overexpressing tumor cells compared to unmodified nanoparticles, thus demonstrating receptor-mediated internalization [132,168]. The high surface area-to-volume ratio of SeNPs combined with peptide-mediated targeting will create a much higher concentration of selenium in the intracellular environment, resulting in an enhancement of therapeutic efficacy and a decrease in nonspecific uptake into normal tissues [171]. Once endocytosed, SeNPs are delivered into endosomal compartments where they slowly release selenium and their therapeutic cargo [35]. Furthermore, many of the peptide systems used for targeting will also act as cell-penetrating peptides such as TAT and promote endosomal escape, thereby facilitating delivery of the nanoparticles to the cytoplasm and other intracellular organelles [65].

### 7.2. ROS Generation and Oxidative Stress

Reactive oxygen species (ROS) are one of the main ways that SeNPs exert their anti-cancer activity [172]. Cancer cells have higher than normal levels of ROS because of metabolic activity as well as mitochondrial damage [173]. SeNPs can cause cancer cells to produce more ROS than they can tolerate, which leads to oxidative damage and subsequently results in cell death [174].

### 7.3. Mitochondrial Damage

SeNPs are transported within the cell to the mitochondria, where they disrupt normal mitochondrial function by inhibiting the activity of the ETC [175]. This disruption will result in overproduction of O_2_^−^, H_2_O_2_ and -OH throughout the mitochondria [176]. Elevated levels of ROS will damage the mitochondrial membrane, thereby causing mitochondrial membrane depolarization, reducing the mitochondrial membrane potential (ΔΨm), and increasing permeability of the outer mitochondrial membrane [177]. Loss of mitochondrial function will also result in decreased ATP production as well as the leakage of pro-apoptotic factors into the cytoplasm [178]. Numerous studies (breast, lung, liver, colorectal) have documented that selenium nanoparticles significantly induce a decrease in mitochondrial membrane potential in various types of cancer cell lines, which ultimately triggers apoptosis [179].

### 7.4. Apoptosis Pathways

The most well-researched effect of selenium nanoparticle treatment throughout the literature is the induction of apoptosis via its intrinsic (mitochondrial) and extrinsic (death receptor-mediated) pathways in cancer cells [180,181]. The intrinsic apoptotic pathway is triggered by various intracellular stressors, including oxidative stress/damage, DNA damage, and dysfunction of mitochondria. Through the formation of reactive oxygen species (ROS) as a result of selenium nanoparticle treatment, there is an alteration of the Bcl-2 family proteins’ pro-apoptotic and anti-apoptotic protein balance. The extrinsic pathway begins when death receptors (e.g., Fas [CD95], tumor necrosis factor receptor, TRAIL receptors) are activated on the surface of the cell [182]. In some models of cancer, selenium nanoparticles have been shown to increase the expression of both death receptors and their ligands [183]. Ligand binding activates caspase-8, which cleaves executioner caspases directly or activates mitochondrial apoptosis through the cleavage of Bid into truncated Bid (tBid) [184]. The cross-communication between the extrinsic and intrinsic pathways has been shown to enhance apoptotic responses and therefore contribute to the efficient elimination of malignant cells [185].

## 8. Signaling Pathway Modulation

Peptide-functionalized selenium nanoparticles (SeNPs) provide additional signaling pathway modulation through oxidative stress-induced apoptosis and also interfere with many oncogenic pathways that regulate cell growth, survival, migration and metastasizing ability.

### 8.1. EGFR Signaling Inhibition

Epidermal growth factor receptor (EGFR) is often overexpressed in numerous cancers such as breast, cervical, colorectal and lung cancers. EGFR activation leads to increased cell growth, angiogenesis, and resistance to cell death via apoptosis [86]. GE11-functionalized SeNPs can specifically target EGFR-expressing cells and will inhibit EGFR phosphorylation and downstream signaling activity [186]. Inhibition of EGFR signaling reduces cancer cell proliferation and increases the sensitivity of tumor cells to undergo apoptosis due to inhibition of signaling pathways associated with cell survival [187]. The increased intracellular accumulation of targeted delivery via EGFR-binding peptides improves the effectiveness of SeNPs [188].

### 8.2. PI3K/Akt Pathway Suppression

The phosphoinositide 3-kinase (PI3K)/Akt pathway is essential for cell survival due to its role in increasing cell metabolism and growth, as well as protecting cells from undergoing apoptosis [189]. PI3K and Akt hyperactivation is commonly found in aggressive tumors [190]. A study has shown that SeNP treatment significantly reduces the phosphorylation of both PI3K and Akt, resulting in inhibition of downstream signaling pathways that regulate cell survival [191]. Furthermore, downregulation of the PI3K/Akt pathway would result in reduced expression of Bcl-2, increased Bax activation, and caspase-dependent mechanisms of inducing apoptosis [192]. Inhibition of this pathway also contributed to decreasing tumor cell migration and invasion [193].

### 8.3. Regulation of MAPK (Mitogen-Activated Protein Kinase) Signaling

MAPK (mitogen-activated protein kinase) signaling is important for cell growth and adaptation of cells to stress and environmental stimuli [194]. Several studies demonstrate that SeNPs can modify many of the MAPK family members, including ERK, JNK, and p38. Cells exposed to selenium have been shown to frequently activate the JNK and p38 pathways, which are involved in the cell’s response to stress and apoptosis [195]. On the other hand, SeNPs are frequently shown to inhibit ERK signaling, which decreases cancer cells’ ability to proliferate [196]. The coordinated regulation of the three MAPK pathways directs the cellular response from survival to apoptosis [194].

### 8.4. Inhibition of STAT3 (Signal Transducer and Activator of Transcription 3)

The oncogenic transcription factor STAT3 (signal transducer and activator of transcription 3) regulates the transcription of genes that promote cell proliferation, angiogenesis, metastasis, and immune evasion [197]. STAT3 is constitutively activated in a number of human cancers. Several studies have shown that SeNPs inhibit both the phosphorylation of STAT3 and the nuclear translocation of this molecule, thereby inhibiting downstream gene expression, including cyclin D1, survivin, VEGF, and Bcl-2 [198,199,200]. Inhibition of STAT3 signaling also inhibits tumor growth, angiogenic activity, and enhances the response of cancer cells to apoptosis [198,199].

### 8.5. Anti-Metastatic and Anti-Angiogenic Effects

**Figure 3:**
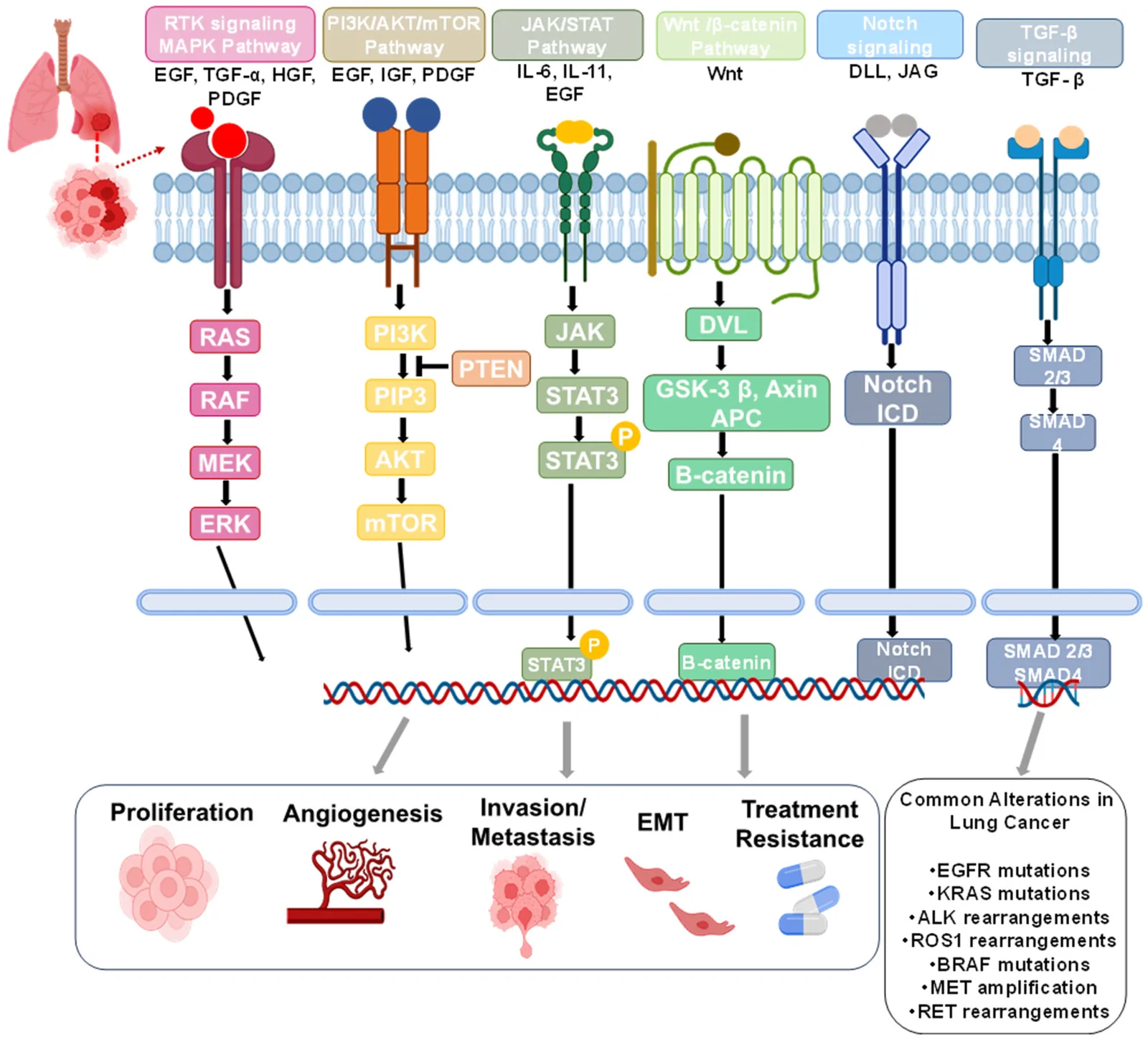
Major oncogenic signaling pathways involved in lung cancer: Aberrant activation of the RTK/MAPK, PI3K/AKT/mTOR, JAK/STAT, Wnt/β-catenin, Notch, and TGF-β/SMAD pathways regulates gene transcription associated with uncontrolled cell proliferation, survival, angiogenesis, epithelial–mesenchymal transition (EMT), metastasis, and therapeutic resistance. These signaling networks represent key molecular targets for peptide-directed nanoparticle-based therapies in lung cancer. Created in BioRender. RAHMANI, A. H. (2026) https://biorender.com/h78e878, accessed on 28 September 2026.

Lung cancer progression is characterized by both widespread metastasis and excessive angiogenesis, both of which have a negative impact on prognosis [130]. One of the primary reasons for the adverse outcomes of lung cancer patients is the presence of metastases and the formation of new vascular networks (angiogenesis) required to support the continuing growth of tumors and their spread to other parts of the body [22]. The results of many studies have shown that selenium nanoparticles (SeNPs) exhibit remarkable anti-metastatic effects by inhibiting migration, invasion, and epithelial–mesenchymal transition (EMT) of cancer cells [130]. It has been scientifically shown that SeNPs inhibit matrix metalloproteinases (MMP-2 and MMP-9), decrease focal adhesion kinase (FAK) signaling, and disrupt the cytoskeletal reorganization of cancer cells, thereby limiting the ability of cancer cells to migrate [201]. SeNPs also induce oxidative stress, which in turn downregulates transcription factors that play a role in metastasis, including NF-κβ and STAT3 [202]. Angiogenesis refers to the development of new blood vessels from pre-existing blood vessels [22]. The formation of new vascular networks is critical to tumor growth and spread to other parts of the body [127]. SeNPs have been shown to inhibit the angiogenic responses of endothelial cells by inhibiting the expression of vascular endothelial growth factor (VEGF), hypoxia-inducible factor-1α (HIF-1α) and their downstream indirect signaling pathways through modulation of the PI3K/Akt/mTOR and MAPK signaling pathways, thereby reducing the proliferation of endothelial cells and the formation of neo-vessels [4]. Pathways involved in lung cancer and the effect of these on proliferation, angiogenesis, metastasis and invasion are illustrated in Figure 3. Recent reviews of selenium nanomedicine for lung cancer have shown that engineered SeNPs disrupt the vascular networks of tumors, while producing less systemic toxicity than conventional anti-angiogenic drugs. Through the combined use of phytochemicals and selenium nanoparticles (SeNPs), the primary potential benefit is related to the inhibition/control of metastasis, specifically through the inhibition of epithelial–mesenchymal transition (EMT), vascular endothelial growth factor (VEGF) production, inflammatory mediator production, and enhanced efficacy in targeting the tumor. The ability of SeNPs to accumulate in tumor tissue due to the use of peptide-targeting ligands also creates greater concentrations within the tumor and provides greater suppression of the hallmark metastatic signaling pathways.

## 9. Synergistic Therapeutic Effects of Peptide-Directed Phytochemical-Coated Selenium Nanoparticles

The enhanced anti-cancer efficacy of phytochemical-coated, peptide-directed SeNPs could be based on the combination of functionalization of three different components (selenium nanoparticle core, phytochemical coating, and peptide-targeting ligand) [35]. However, it is still not fully understood how these three components could interact with each other in vivo to exert synergistic effects. Most of the studies available in the literature separately describe the anti-cancer activity of SeNPs, phytochemical-conjugated SeNPs, or peptide/ligand-conjugated nanocarriers [119,203,204,205]. Based on the available evidence, SeNPs may cause oxidative stress and apoptosis, whereas certain phytochemicals have been reported to modulate cancer cell survival, inflammatory, and angiogenesis pathways [15,93]. Targeting peptides may help the cells recognize and incorporate the nanoparticles by interacting with the corresponding receptors; however, such effects were dependent on the specific types of nanoparticles and respective peptide–receptor pairs [206,207]. Nevertheless, the combined anti-cancer activity of these three components still needs to be experimentally confirmed [93]. Multidrug resistance (MDR) is a common occurrence during cancer treatment that could lead to treatment failure and increased mortality [208]. It is characterized by enhanced drug efflux, DNA repair, and survival signaling pathway activation [4]. Peptide-mediated targeting can increase the association of SeNPs with cells and their subsequent intracellular accumulation through the formation of receptor-mediated interactions, while selenium-induced oxidative stress can disrupt mitochondrial function and cellular bioenergetics [19,49,58,209]. Additionally, a number of phytochemicals such as quercetin, curcumin, berberine and resveratrol have been reported to modulate molecular pathways associated with MDR, including PI3K/Akt, NF-κB and STAT3 signaling [93,210]. Therefore, the application of the aforementioned strategy in vitro and in vivo in cancer models with acquired MDR is of great interest; however, the experimental confirmation of the assumption that the application of all three components together could reverse MDR or increase the efficacy of conventional chemotherapeutics against resistant cancers is still needed.

### 9.1. In Vitro Evidence

Peptide-conjugated SeNPs have not been studied extensively in A549 cells, especially regarding the application of the conjugate of phytochemical functionalization [58]. Studies on other ligand-targeted nanocarriers revealed an improved association of the nanoparticles with cells and their subsequent endocytosis through specific mechanisms [58]. Nevertheless, such data cannot be extrapolated to argue that targeting A549 cells with peptides might bring about the same effect when applied to phytochemical-coated SeNPs. Therefore, it is essential to carry out an experiment that would compare the contribution of targeting ligands to the extent of nanocarrier uptake and the degree of their impact on the capacity of the nanoparticles against cancer cells. Importantly, it has been found that RGDfC-functionalized SeNPs with doxorubicin exhibit enhanced uptake into A549 cells via clathrin-mediated endocytosis, thereby indicating that the uptake of SeNPs guided by peptides is an experimentally achievable process at least in this cell model, but such information is still unavailable for phytochemical-coated SeNPs [58].

IC50, which depends on the plant used as the source of phytochemicals, the size of the nanoparticles, the synthesis process, and surface modification, has been reported to be 43.21 μg/mL for Drimia indica-derived SeNPs [54] and 6.14 μg/mL for Sargassum fusiforme polysaccharide-stabilized SeNPs [211]. Surface modification by phytochemicals increases the cytotoxic effect of the nanoparticles by blocking several oncogenic signals like EGFR, PI3K/Akt, STAT3, and NF-κβ pathway, along with inhibiting inflammation and oxidative stress in the tumor microenvironment. Peptide-conjugated SeNPs were not studied in detail with regard to interaction with A549 cells. However, studies using ligand targeting receptors may show high uptake and accumulation of drugs inside cells.

The H1299 cell line, marked by a lack of functional p53 gene, can be regarded as an ideal test system for studying the selenium-induced cell death pathways unrelated to p53 gene activity. It is especially relevant in view of the high frequency of TP53 mutations in aggressive lung cancers and related drug resistance.

Experimental studies on SeNPs and SeNPs functionalized with phytochemicals or ligands showed the activation of the mitochondrial apoptotic pathway that includes ROS production, loss of mitochondrial membrane potential, cytochrome-c release, caspase-9 and caspase-3 activation, and increased Bax/Bcl-2 ratio [212]. Thus, it was found that the use of selenium-based nanostructures has the ability to induce apoptosis through alternative non-p53-dependent mechanisms. However, it is still unknown whether this type of mechanism also applies to other types of SeNPs coated with peptides or phytochemicals.

H460 large-cell lung carcinoma cells have been widely used for testing the effect of selenium nanoparticles on the process of tumor development and metastasis. Phytochemical-functionalized SeNPs significantly decrease cell viability, the ability to form colonies, migration, and invasion while inducing apoptosis through the process of oxidative stress [212].

It should be noted that the functionalization of the surface of nanoparticles can affect their uptake, intracellular retention, and subsequent biological effects. In experiments with phytochemical-coated or ligand-conjugated SeNPs, an increase in the levels of ROS, the regulation of EMT-associated processes, and the expression of MMP-2 and MMP-9, as well as the pro-angiogenic factor VEGF, were described. Thus, it is possible to propose a hypothesis of the anti-metastatic potential of functionalized SeNPs, although the question of the simultaneous introduction of a targeting peptide and phytochemical coating and the resulting synergistic effect on the inhibition of metastasis have not been answered.

Although several cell lines including A549, H1299, and H460 are still widely used for NSCLC research, other models such as H1975, PC9, and Lewis lung carcinoma (LLC1) have been utilized to study molecularly diverse features of NSCLC. H1975 and PC9 cells can be used to determine the impact of EGFR on NSCLC cells and resistance mechanisms, respectively. The mechanism of selenium nanoparticles conjugated with different phytochemicals and their findings in different cell lines are listed in Table 4. Therefore, these cells could be considered for further investigation of targeting peptides linked to SeNPs. However, the choice of a single targeting moiety should be based on the expression and accessibility of the cognate receptor, and should not be based only on the molecular attributes of the cell line.

### 9.2. In Vivo Evidence

A549-derived xenograft models have been employed to assess the antitumor efficacy and preliminary toxicity of SeNP-based formulations in vivo, with recent studies reporting that SeNPs functionalized or stabilized with bioactive components could exhibit antitumor activity in xenograft models, being also associated with enhanced apoptosis and alterations in intracellular signaling pathways [212,213]. For instance, mitochondrion-targeted SeNPs stabilized with Sargassum fusiforme polysaccharides were found to inhibit A549 xenograft tumor growth in vivo and were associated with ROS production, mitochondrial damage, increased Bax/Bcl-2 ratio, caspase activation, and PI3K/AKT pathway suppression [212], while a bioactive-component-modified SeNP formulation was found to significantly suppress lung tumor growth in vivo, suggesting the potential of functionalized SeNPs as experimental anti-cancer agents [213]. However, it is important to note that both studies investigated the effect of individual SeNP-based formulations and did not examine the combined contribution of a targeting peptide, phytochemical coating, and SeNP core; thus, such findings should be taken as supportive preclinical evidence for the biological activity of functionalized SeNPs rather than conclusive evidence for the cooperative or synergistic activity of all three components.

The antitumor efficacy of SeNP-based formulations reported in the studies was associated with induction of apoptosis, oxidative stress, mitochondrial dysfunction, and alteration of survival signaling pathways [212,213]. In addition, several studies have investigated the effect of SeNP formulations on the development of histopathological changes and general tolerability after treatment, with no significant abnormalities being reported in the experimental animals under the specific experimental conditions [212]. However, it is important to note that such findings pertain to the specific formulations and doses employed in each study, and cannot be extrapolated to other SeNP systems, as the biological and toxicological features of nanoparticles can vary depending on their composition, physicochemical properties, dose, route of administration, and duration of exposure. Thus, in order to assess the safety of peptide-directed, phytochemical-coated SeNPs, additional systematic studies investigating their pharmacokinetics, biodistribution, and toxicological features are required before their therapeutic potential can be determined.

## 10. Challenges in Clinical Translation

Despite favorable preclinical results, the transition of peptide-conjugated phytochemical-coated selenium nanoparticles (SeNPs) into the clinic is limited due to the variety of scientific, technological and regulatory issues [215]. One of the main obstacles to the advancement of this approach lies in the lack of established protocols for synthesis [216]. Diverse protocols used for nanoparticle synthesis, phytochemical selection, and peptide conjugation and purification often result in nanoparticles with different size, morphology, surface chemistry, peptide loading capacity, and biological activity, thus making the replication of results difficult [61].

The second important issue that has to be addressed in developing a therapy with SeNPs is the dosing regimen [217]. Given the limited therapeutic index of selenium, optimal doses and schedules have to be carefully determined, and comprehensive toxicology assessment, including chronic toxicity, immunogenicity, organ accumulation, biodegradability and environmental impact, should be carried out before applying to the clinic [218,219].

Pharmacokinetic information is also insufficient. It is unknown how peptide-functionalized selenium nanoparticles are absorbed, distributed, metabolized and excreted in an organism, and what happens in terms of forming a protein corona [220]. Rapid recognition by the mononuclear phagocyte system limits the circulation time and accumulation in the tumor site; therefore, new approaches to improving nanoparticles’ stability and systemic circulation are needed [221].

Regulatory issues constitute another barrier. Complex physical and chemical properties of multifunctional nanoparticles require stringent quality control measures, scalable production under good manufacturing practice conditions, and standardization of characterization protocols. In addition to these, peptide stability at storage and large-scale peptide conjugation are important concerns [12,222].

## 11. Current Research Gaps

In spite of encouraging anti-cancer results obtained with SeNPs and phytochemical-functionalized SeNPs in vitro and in vivo, little research has been conducted regarding the combination of selenium nanoparticles, phytochemicals and targeting peptides in one system. The majority of the available studies focused either on phytochemical-functionalized SeNPs or on peptide/ligand-conjugated nanoparticles. Therefore, the suggested three-component design should initially be treated as a concept based on reasoning and generating hypotheses, rather than as a proven system of synergistic action. Studies comparing the effect of individual components and the combination of all components are needed to assess whether a synergistic, additive or no-effect relationship exists.

Another key limitation is the lack of standardized characterization of their physicochemical and surface properties. The characteristics of particle size distribution, zeta potential, phytochemical loading, peptide density and orientation, conjugation efficiency, colloidal stability, and surface composition are rarely reported in detail. Such variability between formulations may explain differences in their biological responses. The establishment of standardized criteria for characterization and reporting would enable more consistent development and facilitate the transition from promising formulations to preclinical evaluation.

Finally, the molecular mechanisms of the action of multifunctional SeNPs are still poorly understood. The biological activities of these NPs may depend on the types and oxidation states of the contained Se species, as well as the size and composition of the NPs, the type of phytochemicals and their content in the NPs, the characteristics of the interaction between NPs and cells, the pathways of intracellular transport of the NPs and phytochemicals, and the formation of protein coronae on the surface of the NPs. Since these various factors can be interdependent, it is difficult to separate and quantify their individual contributions to the observed effects. To fully understand the modes of action, a combination of integrated “omics” methods, mechanistic studies, and appropriate animal models is needed to reveal the individual and combined contributions of selenium, phytochemicals, and targeting moieties.

## 12. Future Perspectives and Conclusion

Future research directions should be focused on the creation of multifunctional SeNP-based platforms, which will allow the implementation of precision and personalized nanomedicine. Innovations in molecular profiling can contribute to the identification of peptides that will match the specific receptors expressed in patients, thus providing more effective targeting of tumors and improved therapeutic results with minimal toxicity. Combination therapy appears to be an interesting strategy for using peptide-mediated phytochemical-coated nanoparticles. Due to their multifunctional surface, such nanoparticles can transport different types of cargo, including conventional chemotherapeutic drugs, phytochemicals, nucleic acids, immune modulators, or gene-editing molecules in a single nanoplatform. This approach will help to address drug resistance issues, stimulate antitumor immunity, and modulate several oncogenic pathways simultaneously. The use of stimuli-responsive or responsive nanoparticles able to release therapeutics exclusively within the tumor microenvironment can also be considered a promising strategy.

Besides the mentioned opportunities, several challenges are associated with the clinical translation of tumor-targeting peptide-functionalized SeNPs, including low tumor accumulation due to rapid biodistribution and clearance by the mononuclear phagocyte system, tumor heterogeneity and diversity of receptor overexpression that may hinder the utilization of a single targeting ligand, inefficient endosomal escape after receptor-mediated internalization, and uncontrolled release of therapeutic agents without achieving the intended intracellular location. In this regard, current research may be focused on reducing the size of nanoparticles and optimizing their surface chemistry, minimizing the ligand density and undesirable orientation on the nanoparticle surface, engineering suitable linkers, strategies for endosomal escape, and stimuli-responsive ligand release. In addition, the development of multifunctional formulations that incorporate various tumor-targeted ligands and different types of drugs, including phytochemicals, chemotherapy, nucleic acids, or immunomodulatory agents, might be considered. At the same time, the complexity of the formulation and the necessity to balance multiple variables should be taken into account when designing drug delivery systems. The tumor molecular profile and expression pattern of targeting receptors may also be integrated into the design of novel nanomedicines, enabling the selection of the most appropriate ligands and combination of therapeutic agents for each patient. However, such approaches require extensive preclinical studies, including pharmacokinetics, toxicity, and clinically relevant efficacy evaluations.

Further research will need standardization of the synthesis protocols, pharmacokinetics and toxicology studies, clinically relevant animal models, and well-designed human clinical trials. Solving these issues will play a key role in the translation of peptide-guided phytochemical-coated SeNPs from experimental models to clinical applications.

## Figures and Tables

**Table 1:** **Clinically relevant molecular targets in NSCLC and their potential relevance to peptide-directed SeNP therapy:** Major oncogenic receptors and signaling pathways involved in NSCLC progression, their biological functions, and their potential relevance to peptide-mediated targeting and SeNP-based therapeutic strategies.

S. No.	Molecular Target/Pathway	Role in NSCLC	Therapeutic Relevance	Potential Peptide-Targeting Strategy	Relevance to SeNPs	Ref.
1.	EGFR	Promotes proliferation, survival, migration and therapy resistance	Major therapeutic target, particularly in EGFR-altered NSCLC	EGFR-binding peptides such as GE11	Peptide-mediated uptake may enhance intracellular SeNP delivery and complement inhibition of EGFR-associated signaling	[94,95,96]
2.	KRAS	Drives MAPK and PI3K/AKT signaling and contributes to aggressive tumor behavior	Important driver of NSCLC and therapeutic resistance	Indirect targeting through peptides directed toward surface receptors associated with KRAS-driven tumors	SeNP-induced oxidative stress may interfere with downstream survival signaling	[95,97,98]
3.	ALK	Aberrant ALK signaling promotes proliferation and survival	Important oncogenic driver in a subset of NSCLC	ALK-associated targeting strategies	Potential for targeted delivery to ALK-positive tumor populations	[95,98,99]
4.	PI3K/AKT/mTOR	Regulates cell survival, proliferation, metabolism and resistance	Major downstream therapeutic pathway	Peptide-mediated receptor targeting rather than direct pathway binding	SeNPs and phytochemicals may modulate this pathway and enhance apoptosis	[94,96,97]
5.	MAPK/ERK	Regulates proliferation, differentiation and survival	Frequently activated downstream of EGFR/KRAS	Receptor-targeting peptides	ROS-mediated signaling effects of SeNPs may suppress downstream proliferative signaling	[96,100]
6.	VEGF/VEGFR	Promotes angiogenesis and tumor progression	Relevant for tumor vascularization and metastasis	RGD/iRGD or VEGFR-targeting peptides	Peptide targeting may improve tumor localization while SeNPs/phytochemicals exert anti-angiogenic effects	[96,100]
7.	Integrins (αvβ3/αvβ5)	Regulate adhesion, migration, invasion and angiogenesis	Useful surface targets for tumor and tumor-vasculature targeting	RGD/iRGD peptides	Can enhance tumor-associated uptake and potentially reduce nonspecific distribution	[96,97]
8.	STAT3	Supports survival, inflammation, immune evasion and metastasis	Important therapeutic signaling node	STAT3-associated or receptor-targeting peptides	Phytochemicals and SeNPs may suppress STAT3-associated signaling	[96,97]
9.	HER2	Promotes proliferation and survival in a subset of tumors	Potential target in HER2-expressing NSCLC	HER2-binding peptides	Provides an additional receptor-mediated targeting option	[95,98,99]

**Table 3:** **Peptide ligands and molecular targets relevant to targeted selenium nanoparticle delivery in NSCLC:** Tumor-targeting peptides, their corresponding receptors, mechanisms of cellular targeting, and their potential utility for improving the selective delivery and intracellular uptake of phytochemical-coated SeNPs in NSCLC.

S. No.	Peptide	Target/Receptor	Targeting Mechanism	Potential Application in SeNP Delivery	Ref.
1.	GE11	EGFR	Receptor-mediated binding/internalization	Targeting EGFR-overexpressing NSCLC cells	[148,149]
2.	RGD	αvβ3/αvβ5 integrins	Integrin-mediated targeting	Tumor/angiogenic vasculature targeting	[150]
3.	iRGD	Integrins/neuropilin-associated pathway	Tumor penetration and intracellular delivery	Enhanced tumor penetration	[81,151,152]
4.	NGR	CD13	Receptor-mediated targeting	Tumor-associated vasculature targeting	[153,154,155]
5.	Bombesin-derived peptides	GRPR	Receptor-mediated uptake	Targeting GRPR-expressing tumors	[145,147]
6.	CXCR4-targeting peptides	CXCR4	Receptor-mediated targeting	Potential targeting of CXCR4-positive tumor cells	[156,157]

**Table 4:** Selenium nanoparticles conjugated with different phytochemicals with their role against different cancer cell lines, their mechanism.

S. No.	Cell Lines/Model	SeNP Formulation	Major Mechanism	Key Findings	Ref.
1	A549	Biosynthesized SeNPs	ROS generation, mitochondrial apoptosis, caspase-3 activation	Significant inhibition of proliferation with increased apoptosis	[128]
2	A549	*Drimia indica*-mediated SeNPs	ROS, p53 activation, G2/M arrest	Induced apoptosis and cell-cycle arrest	[22]
3	A549	*Sargassum wightii*-derived SeNPs	ROS-mediated apoptosis, AKT modulation	Reduced viability and increased apoptotic signaling	[131]
4	A549	Plant-derived SeNPs	Bax/Bcl-2 regulation, caspase activation	Enhanced apoptosis with minimal toxicity to normal cells	[130]
5	H1299	Functionalized SeNPs	p53-independent mitochondrial apoptosis	Cytochrome-c release and activation of caspase-9/-3	[22]
6	H460	Surface-functionalized SeNPs	ROS, inhibition of migration and invasion	Reduced colony formation and metastatic behavior	[22]
7	A549 + X-ray	SeNPs + radiotherapy	Radiosensitization, enhanced caspase activation	Greater apoptosis than SeNPs alone	[4]
8	NSCLC	SeNP-based nanomedicine	Drug delivery, immunomodulation	Improved therapeutic efficacy with reduced systemic toxicity	[22]
9	Lung cancer tumor bearing mice	AANL-SeNPs	Upregulation of CLK2	Significant suppression of tumor growth; tumor size was approximately 79% lower in the 2 mg/kg AANL-SeNP group than in the PBS group	[213]
10	tumor bearing mice	AAGL-SeNPs	Upregulation of KLF4, Immune cell mediated antitumor activity	Suppressed lung tumor growth and enhanced antitumor activity; reported reduction in AAGL-associated hepatotoxicity	[214]

## Data Availability

No new data were created or analyzed in this study. Data sharing is not applicable to this article.

## Abbreviations

**A549**—Human lung adenocarcinoma cell line; **AKT/Akt**—Protein kinase B; **ALK**—Anaplastic lymphoma kinase; **ATP**—Adenosine triphosphate; **Bax**—BCL2-associated X protein; **Bcl-2**—B-cell lymphoma 2; **BSA**—Bovine serum albumin; **CAFs**—Cancer-associated fibroblasts; **CD95**—Cluster of differentiation 95 (Fas receptor); **CRISPR/Cas**—Clustered regularly interspaced short palindromic repeats/CRISPR-associated protein; **CXCR4**—C-X-C chemokine receptor type 4; **CXCL12**—C-X-C motif chemokine ligand 12; **DNA**—Deoxyribonucleic acid; **ECM**—Extracellular matrix; **EDC**—1-Ethyl-3-(3-dimethylaminopropyl)carbodiimide; **EDC/NHS**—1-Ethyl-3-(3-dimethylaminopropyl)carbodiimide/N-hydroxysuccinimide coupling system; **EGF**—Epidermal growth factor; **EGFR**—Epidermal growth factor receptor; **EMT**—Epithelial–mesenchymal transition; **EPR**—Enhanced permeability and retention; **ERK**—Extracellular signal-regulated kinase; **ETC**—Electron transport chain; **FAK**—Focal adhesion kinase; **Fas**—Fas cell-surface death receptor; **G2/M**—G2/M phase of the cell cycle; **GE11**—Synthetic EGFR-binding peptide (YHWYGYTPQNVI); **GLOBOCAN**—Global Cancer Observatory; **GRB2**—Growth factor receptor-bound protein 2; **GRPR**—Gastrin-releasing peptide receptor; **H1299**—Human non-small-cell lung cancer cell line; **H1975**—Human lung adenocarcinoma cell line; **H460**—Human large-cell lung cancer cell line; **HER2**—Human epidermal growth factor receptor 2; **HIF-1α**—Hypoxia-inducible factor 1-alpha; **IC50**—Half-maximal inhibitory concentration; **IL-6**—Interleukin-6; **IL-10**—Interleukin-10; **iRGD**—Internalizing RGD peptide; **JAK**—Janus kinase; **JAK/STAT**—Janus kinase/signal transducer and activator of transcription pathway; **JNK**—c-Jun N-terminal kinase; **KRAS**—Kirsten rat sarcoma viral oncogene homolog; **LC**—Lung cancer; **LLC1**—Lewis lung carcinoma 1 cell line; **MAPK**—Mitogen-activated protein kinase; **MDR**—Multidrug resistance; **MDSCs**—Myeloid-derived suppressor cells; **MET**—MET proto-oncogene, receptor tyrosine kinase; **MMP-2**—Matrix metalloproteinase-2; **MMP-9**—Matrix metalloproteinase-9; **mTOR**—Mechanistic target of rapamycin; **M**—Classically activated/pro-inflammatory macrophage phenotype; **M2**—Alternatively activated/immunosuppressive macrophage phenotype; **Na_2_SeO_3_**—Sodium selenite; **NaBH_4_**—Sodium borohydride; **NF-κB** —Nuclear factor kappa B; **NH_2_**—Amino group; **NGR**—Asn-Gly-Arg tumor-homing peptide; **NGR-hTNF**—NGR-targeted human tumor necrosis factor; **NHS**—N-Hydroxysuccinimide; **NPs**—Nanoparticles; **NSCLC**—Non-small-cell lung cancer; **OH**—Hydroxyl group; **p38**—p38 mitogen-activated protein kinase; **p53**—Tumor protein p53; **PC9**—Human lung adenocarcinoma cell line; **PD-1**—Programmed cell death protein 1; **PD-L1**—Programmed death-ligand 1; **PEG**—Polyethylene glycol; **pH**—Potential of hydrogen; **PI3K**—Phosphoinositide 3-kinase; **PI3K/AKT/mTOR**—Phosphoinositide 3-kinase/protein kinase B/mechanistic target of rapamycin pathway; **PVA**—Polyvinyl alcohol; **Ras**—Rat sarcoma family of small GTPases; **RGD**—Arg-Gly-Asp peptide/motif; **RGDfC**—RGD-containing cyclic peptide containing phenylalanine and cysteine; **ROS**—Reactive oxygen species; **RTK**—Receptor tyrosine kinase; **RTK/MAPK**—Receptor tyrosine kinase/mitogen-activated protein kinase pathway; **Se**—Selenium; **SeNP**—Selenium nanoparticle; **SHC**—Src homology 2 domain-containing transforming protein C; **siRNA**—Small interfering RNA; **STAT3**—Signal transducer and activator of transcription 3; **T790M**—Threonine-to-methionine substitution at codon 790 of EGFR; **TAMs**—Tumor-associated macrophages; **TAT**—Trans-activator of transcription peptide; **TGF-β/SMAD**—Transforming growth factor-beta/SMAD signaling pathway; **TKIs**—Tyrosine kinase inhibitors; **TME**—Tumor microenvironment; **TP53**—Tumor protein p53 gene; **TRAIL**—TNF-related apoptosis-inducing ligand; **Tregs**—Regulatory T cells; **tBid**—Truncated BH3-interacting domain death agonist (truncated Bid); **UCRD**—University Center for Research & Development; **VEGF**—Vascular endothelial growth factor; **VEGFR**—Vascular endothelial growth factor receptor; **VEGFR-2**—Vascular endothelial growth factor receptor 2; **YHWYGYTPQNVI**—Amino-acid sequence of the GE11 peptide; **αvβ-3/5**—Integrin alpha-v beta-3/5; **Wnt/β-catenin**—Wingless-relate/d integration site/beta-catenin signaling pathway.
